# Assessment of QRS duration and presence of fragmented QRS in patients with celiac disease

**DOI:** 10.7150/ijms.98131

**Published:** 2024-07-16

**Authors:** İbrahim Ethem Güven, Mustafa Candemir

**Affiliations:** 1 Department of Gastroenterology, Ankara Yildirim Beyazit University Yenimahalle Training and Research Hospital, Ankara, Turkey.; 2 Department of Cardiology, Gazi University School of Medicine, Ankara, Turkey.

**Keywords:** cardiac arrhythmia, electrocardiogram, fragmented QRS, QRS duration, celiac disease

## Abstract

**Background:** Celiac Disease (CD) is characterized by small intestine involvement. However, cardiac manifestations may also be seen in the clinical course. The significance of the QRS prolongation and the presence of QRS fragmentation (fQRS) has been previously studied in many chronic inflammatory disorders as an independent predictor of cardiac manifestations. The study aimed to evaluate the QRS duration and presence of fQRS in patients with CD.

**Methods:** 164 patients with CD and 162 healthy controls were included in the present study. QRS duration and presence of fQRS were calculated from the 12-lead electrocardiogram and compared between groups. The association between these parameters and disease duration was also evaluated.

**Results:** QRS duration was found to be higher in the CD group compared to the control group (83 (76.8-93) vs. 91 (84-94), p<0.001). The presence of fQRS was demonstrated to be higher in the CD group (n=68 (41.5%) vs n=42 (25.9%), p=0.003). Notably, QRS duration was positively correlated with disease duration (Spearman's Rho= 0.47, p<0.001). In addition, disease duration was significantly higher in the fQRS (+) group (60 (23,5-144) vs. 28,5 (15-71,5), p=0.002).

**Conclusion:** This study revealed that QRS prolongation and the presence of fQRS were higher in patients with CD. The presence of these findings may be an indicator of early subclinical cardiac involvement, especially in those with long disease duration. Thus, patients with these ECG findings can be considered for further cardiac evaluation.

## Introduction

Celiac Disease (CD) is a chronic inflammatory disorder characterized by an impaired immune response to gluten 1. CD mainly involves the small intestine and usually presents with gastrointestinal symptoms and signs of malabsorption 2. However, inflammation is not restricted to the gastrointestinal tract, and extraintestinal manifestations can also be observed in the course of the disease, including neurological, ocular, dermatologic, musculoskeletal, and cardiovascular manifestations 3. In addition, it has been demonstrated that subclinical inflammation can persist even in cases of adherence to the gluten-free diet 4. Among extraintestinal manifestations, cardiovascular involvement is of great importance as it can be associated with high morbidity and mortality when left unattended 5.

Today, Electrocardiogram (ECG) along with anamnesis still maintains its importance as the first step in the assessment of cardiovascular diseases 6. As the association between chronic inflammatory processes and cardiac clinical implications has been well established in the literature, pathological abnormalities in ECGs in inflammatory systemic diseases have attracted interest in recent years 7. In this concept, prolongation in the QRS duration and the presence of fragmented QRS (fQRS), which are associated with an increased risk of arrhythmia and myocardial fibrosis, respectively, have been widely evaluated in patients with chronic inflammatory systemic disorders 8-10. However, to the best of our knowledge, the significance of fQRS and QRS duration has never been evaluated in patients with CD previously.

In the present study, we aimed to evaluate the QRS duration and presence of fQRS in patients with CD.

## Patients and Methods

### Study populations

This retrospective observational study was conducted in the Gastroenterology department of our tertiary hospital. All eligible patients diagnosed with CD, followed between January 2020 and February 2024, were enrolled in the study. The diagnosis of celiac disease was made on the basis of histopathological findings combined with anamnesis and celiac serology.

All subjects were assessed for eligibility for the study by applying the following exclusion criteria prior to inclusion in the study: i) Younger than 18 years old; ii) History of coronary artery disease; iii) ischemic findings on the ECG; iv) severe valvular disease and left ventricular dysfunction (patients with decreased ejection fraction) on echocardiographic examination; v) history of intraventricular conduction delay, complete bundle branch block patterns (QRS >120 ms), incomplete right bundle branch block, and arrhythmia; vi) hyperthyroidism or hypothyroidism, chronic liver and kidney diseases, chronic respiratory diseases, diabetes mellitus, hypertension, autoimmune diseases; vii) electrolyte abnormalities in laboratory data; viii) malignancy; ix) use of any antiarrhythmic medication (ß-Adrenergic blockers, calcium channel blockers, sodium channel blockers, potassium channel blockers). In addition to the exclusion criteria mentioned above, patients with a diagnosis of CD of less than one year and patients with less than six months of dietary compliance were also excluded from the study. Lastly, in order to make accurate measurements, patients whose ECG image quality was not of sufficient resolution for assessment were excluded from the study.

An age- and gender-matched control group was recruited prospectively from consecutive patients who applied to our institution's outpatient clinic with complaints of dyspepsia and without any pathological findings during the further evaluation phase. Before inclusion in the control group, it was confirmed by anamnesis that they had no underlying disease and were not taking any medication. In addition, exclusion criteria were also applied to the control group.

Ethical approval was retrieved from Ankara Training and Research Hospital Scientific Research Assessment and Ethics Committee (Approval No: E-24-93).

### Data collection and electrocardiographic assessment

Demographic data such as age, gender, disease duration and body mass index (BMI) were collected through electronic and printed medical records in the hospital data system. Detailed anamnesis of the patients was obtained from the patient interview forms recorded at each outpatient clinic visit. The Laboratory results of the CD patients and the control group, including hematological and biochemical profiles, were recorded from hospital digital records.

12-lead ECG records of both the CD patients and the control group were obtained from the hospital's data archive. A recorder set at a 25 mm/s paper speed and a voltage calibration of 1 mV/cm was used in the assessment. The subjects ' ECG data were scanned and transported to a computer in a high-resolution document format. In order to ensure sensitive and accurate measurements, the ECG recordings were magnified 600 times during the evaluation phase. All assessments were carried out by a single Cardiologist who was blinded to the patient's data throughout the evaluation process. QRS duration was measured by calculating the interval from the onset of the Q wave to the end of the S wave. The longest QRS in any lead was selected for evaluation. The definition of fQRS was based on the presence of the following ECG features: The appearance of an additional R wave (R') or notching in the nadir of the R wave or the S wave, or the presence of fragmentation (at least more than one R') in 2 contiguous leads.

### Statistical analysis

The IBM Statistical Package for the Social Sciences Statistics for Windows, version 25.0 (IBM Corp., Armonk, NY, USA), was used for analysis. The normality distribution of numerical variables was evaluated by using the Kolmogorov-Smirnov test. Numerical variables were compared using the Independent Samples T Test or the Mann-Whitney U test, which was appropriate. Categorical variables were compared by using the Chi-square test. The correlation between disease duration and QRS interval was evaluated using Spearman's test. Multivariable logistic regression analyses were performed to determine the independent predictors of the presence of celiac disease. A 2-sided P <0.05 was considered significant.

## Results

A total of 326 participants, including 164 patients with CD and 162 subjects in the control group, were included in the study. The median age was 40 (26-49) years in the CD group and 41.5 (31-51.3) years in the control group. The female gender ratio of the CD patients and the control group were 69.5% (n=114) and 62,3% (n=101), respectively. Detailed demographic data, laboratory, and 12-lead ECG findings of the participants are presented in Table 1. Group comparison of the CD and control group revealed no significant differences regarding age, gender, smoking, BMI, and laboratory data (for all parameters, p<0.005). The median disease duration of the CD group was 39 (16.3-96) months.

**Figure 1:**
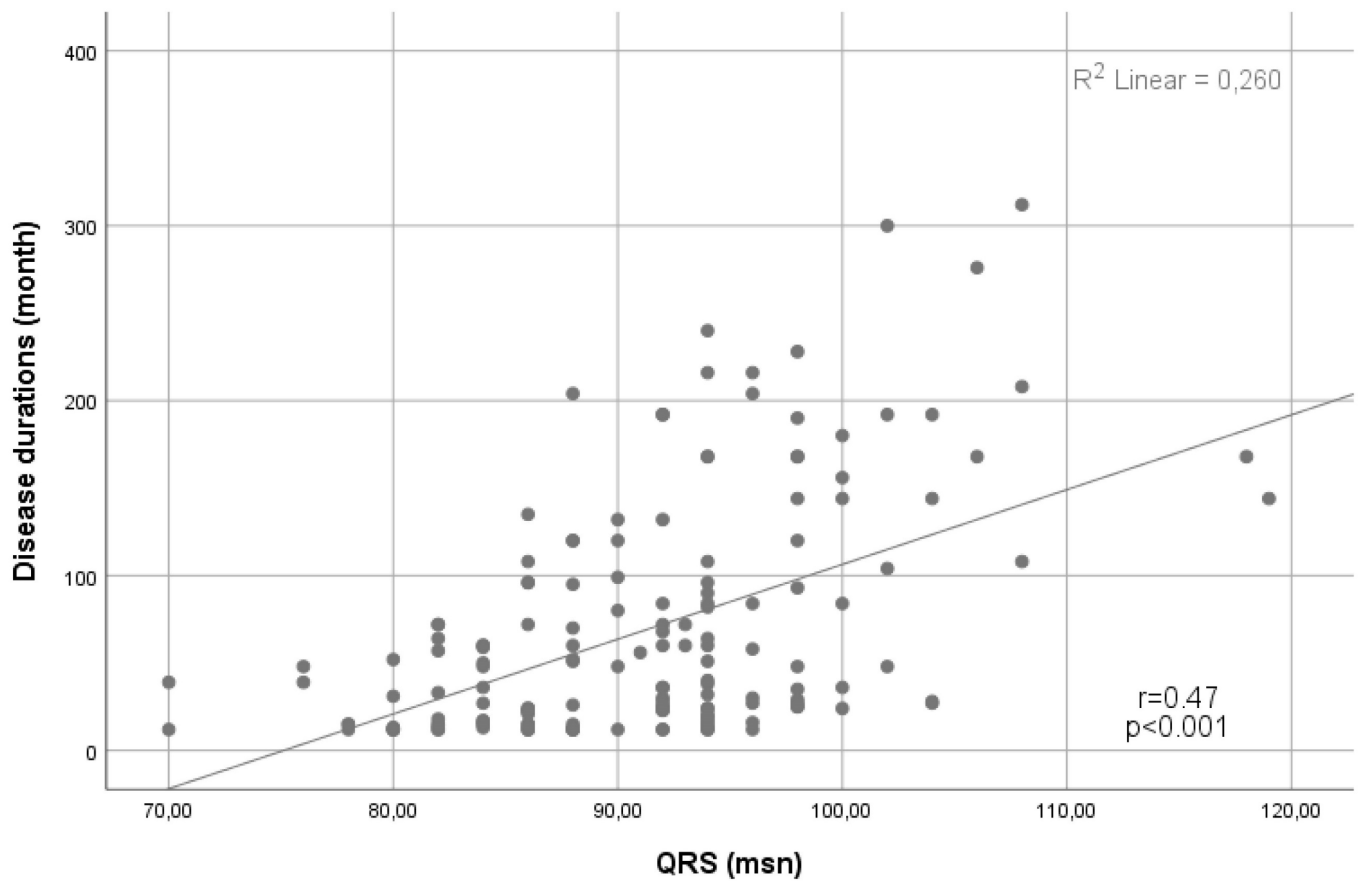
Scatter plot showing a positive linear correlation between QRS duration and disease duration.

In terms of the ECG findings, the median QRS interval was higher in the CD group than in the control group (83 (76.8-93) vs. 91 (84-94), p<0.001). In addition, the presence of fQRS was significantly higher in the CD group (n=68 (41.5%) vs n=42 (25.9%), p=0.003) (Table 1). Notably, a positive correlation was demonstrated between the QRS duration and the disease duration (Spearman's Rho= 0.47, p<0.001) (Figure 1).

Univariate and multivariable logistic regression analyses of predictors for CD were revealed in Table 3. QRS interval and fQRS were the independent predictors of the CD in multivariable logistic regression analyses (for all parameters, p<0.005) (Table 2).

When IBD patients were divided into two groups on the basis of the presence of fQRS as fragmented QRS (+) and fragmented QRS (-), no difference was found in group comparisons regarding demographic characteristics and laboratory data. However, disease duration was higher in the fQRS (+) group than in the fQRS (-) group (60 (23,5-144) vs. 28,5 (15-71,5), p=0.002) (Table 3).

## Discussion

In the present study, we have demonstrated that, although within the normal reference limits, QRS duration was longer, and the presence of fQRS was higher in patients with CD. Noteworthy, a positive correlation was demonstrated between the disease duration and the QRS duration. In addition, disease duration was also higher in the fragmented QRS (+) group than in the fragmented QRS (-) group.

CD is a chronic inflammatory disorder that affects approximately 0.5-1% of the population and can occur at any age 11. CD predominantly involves the small intestine and is characterized by gastrointestinal symptoms and signs and symptoms of malabsorption 12. However, with the increased knowledge about CD, it has been demonstrated that the inflammatory process is not limited to the gastrointestinal system and can be accompanied by extraintestinal manifestations 13. Among these, cardiac manifestations have recently become of increasing interest since they are associated with increased mortality and morbidity when unaddressed 14, 15. An increased risk of ischemic heart disease in patients with CD was demonstrated in several previous studies 16, 17. In addition, CD was found to be associated with an increased risk of impaired left ventricular dysfunction, cardiomyopathy, and heart failure 18, 19. Moreover, CD patients have been shown to have conduction disturbances secondary to interatrial mechanical delay and are at risk for the development of atrial fibrillation 20. It is also noteworthy that cardiac pathophysiological changes, including subclinical myocardial dysfunction and subclinical cardiac fibrosis, were shown to be detectable even in the pediatric population in previous studies 21, 22. The underlying mechanism of cardiac manifestations is mainly secondary to pathophysiological changes facilitated by the chronic inflammatory state 13. Increased pro-inflammatory cytokines secondary to the activated immune system induce the development of atherosclerosis and predispose to the development of myocardial fibrosis 23. Increased levels of endothelin and procoagulant factors lead to the development of endothelial dysfunction 24. In addition, increased oxidative stress, which is a consequence of the chronic inflammatory process, also contributes to these pathophysiological changes by causing subclinical ischemic alterations 25. The cumulative damage caused by the above-mentioned processes over time leads to fibrinogenic remodeling of cardiomyocytes, which can result in impaired cardiac function. These pathophysiological changes can lead to an increased risk of arrhythmias since the cardiac conduction system may also be affected by the mentioned fibrinogenic remodeling 26.

QRS duration is an indicator of ventricular depolarization, and prolongation in QRS duration has been shown to be associated with an increased risk of arrhythmia and cardiac adverse events 27. In a study by Baslaib *et al.*, prolonged QRS duration was found to be an independent predictor of mortality in patients with acute coronary syndromes 28. Similarly, Lund *et al.* demonstrated that QRS duration has a prognostic value in determining the severity of heart failure and was a predictor of mortality 29. In addition, previous studies have demonstrated the relationship between the prolongation in QRS duration and increased risk of ventricular arrhythmia 30. According to the results of this study, it is remarkable that the QRS duration was longer in CD patients compared to the healthy control group. Notably, the fact that prolongation of QRS duration was positively correlated with increased disease duration indicates that the conduction system, which is cumulatively exposed to the chronic inflammatory process in the long term, maybe subclinically impaired. Consistent with the findings in our study, QRS duration has also been shown to be associated with disease duration in many systemic inflammatory diseases 8, 31. Considering that QRS prolongation may be associated with increased arrhythmia risk and cardiovascular events, QRS prolongation in CD patients, especially with long disease duration, is an issue to be addressed in further studies.

Myocardial fibrosis is another consequence of fibrinogenic remodeling facilitated by the chronic inflammatory state. Previous studies have demonstrated that QRS fragmentation is an indicator of subclinical myocardial fibrosis and is associated with adverse outcomes 32. Since the relationship between chronic inflammation and the presence of fQRS has been well established, the significance of fQRS has been widely evaluated in patients with chronic inflammatory diseases. In this concept, studies conducted on Behçet's disease, systemic sclerosis, ankylosing spondylitis, nephrotic syndrome, and rheumatoid arthritis have revealed a higher rate of fQRS presence compared to the control group 8, 33-36. Our findings were consistent with the previous studies, which also demonstrated a higher rate of fQRS in patients with CD than the healthy control group. In addition, when CD patients were compared with each other based on the presence of fQRS, it was found that the fQRS (+) group had a higher disease duration. Since fQRS is a marker for myocardial fibrosis, the fact that the presence of fQRS was more frequent in individuals with longer disease duration again emphasizes the importance of the duration of exposure to a chronic inflammatory state, as previously mentioned. Since disease duration is an important factor in the development of pathophysiological remodeling, clinicians should keep in mind that cardiological evaluation should be performed more sensitively, especially in the follow-up of patients with long disease duration. However, it should also be noted that although these findings have been shown to be associated with adverse cardiovascular events, it cannot be concluded that every case with the mentioned ECG findings will develop these adverse events in the future clinical course. Therefore, further studies, including long-term clinical follow-up, may provide a more comprehensive understanding of QRS duration and the presence of fQRS.

This study has some limitations. Firstly, it was a retrospective study. Therefore, cardiovascular adverse events that may develop during clinical follow-up could not be established. Secondly, the study was a single-center study and had a relatively small sample size. Therefore, multicenter studies with a larger number of subjects are needed. Lastly, sensitive evaluation of myocardial fibrosis by cardiac magnetic resonance imaging could not be provided.

In conclusion, our findings revealed significantly higher QRS duration and the presence of fQRS in patients with CD and a positive correlation between QRS duration and disease duration. Determining the possible risk factors related to the development of cardiac manifestations can contribute to the prevention of cardiac adverse events that may develop in the clinical follow-up of CD. In the presence of these impaired ECG findings, clinicians may consider further cardiological assessment, especially in patients with long disease duration.

## Author Contributions

Study conception and design: İ.E.G. and M.C.; data collection: İ.E.G.; data analysis: İ.E.G. and M.C.; drafting and critical revision of manuscript: İ.E.G. and M.C. All the authors read and approved the final version of the manuscript.

## Figures and Tables

**Table 1:** Baseline characteristics, laboratory and 12-Lead Electrocardiographic parameters of the study participants.

	Celiac disease (n=164)	Control (n=162)	*P*
Age, years	40 (26-49)	41.5 (31-51.3)	0.144
Sex (Female), n (%)	114 (69.5)	101 (62.3)	0.172
Smoking, n (%)	50 (30.5)	45 (27.8)	0.590
BMI, kg/m^2^	25 (23.9-26.3)	25 (23-26.6)	0.311
Glucose, mg/dL	93 (84-110)	96 (87-108.3)	0.284
WBC, ×10^3^/ml	6.9 ± 1.6	7.0 ± 2.1	0.525
Hemoglobin, g/dL	13.5 ± 1.6	13.4 ± 1.8	0.515
AST, U/L	18 (15-23.8)	19.5 (14-29.8)	0.170
ALT, U/L	19 (16-25)	20 (16-25)	0.547
Creatinine, mg/dL	0.7 (0.6-0.9)	0.7 (0.6-0.9)	0.761
Sodium, mmol/L	140 (139-141)	140 (138.8-142)	0.829
Potassium, mmol/L	4.3 (4.1-4.5)	4.2 (3.9-4.5)	0.176
Calcium, mg/dL	9.6 (9.1-9.8)	9.6 (9.2-9.9)	0.604
Total cholesterol, mg/dL	167.9 ± 39.3	168.1 ± 38.2	0.973
Triglyceride, mg/dL	121 (82.5-164.5)	117 (78-160.3)	0.550
HDL, mg/dL	44 (36-55.8)	45 (39-53)	0.430
LDL, mg/dL	92 (72.3-116)	45 (39-53)	0.692
Sedimentation rate, mm/h	8 (5-12)	8 (4-12)	0.923
C-reactive protein, mg/dL	1 (0.5-2.8)	1.2 (0.6-3.2)	0.417
Heart rate, beat/min	78 (69-87)	77.5 (70-88)	0.657
QRS interval, ms	91 (84-94)	83 (76.8-93)	**<0.001**
QTc interval, ms	423 (407-438.8)	425.5 (395.5-460.4)	0.537
fQRS, n (%)	68 (41.5)	42 (25.9)	**0.003**
Disease duration, months	39 (16.3-96)	-	**-**

Results are expressed as: mean ± SD or median (IQR) or frequency (%). SD: Standard deviation, IQR: Interquartile range, BMI: body mass index, WBC: white blood cell, AST: aspartate aminotransferase, ALT: alanine aminotransferase, HDL: high-density lipoprotein, LDL: low-density lipoprotein, QTc: Corrected QT interval, fQRS: Fragmented QRS. Statistically significant results (p<0.05) were shown in bold type.

**Table 2:** Multivariable logistic regression analyses of predictors for Celiac disease.

	Multivariable analysis			
		95% CI		
	OR	Lower	Upper	*P*
Age	0.983	0.966	1.001	0.066
Sex	1.552	0.938	2.569	0.087
BMI	1.003	0.920	1.094	0.945
CRP	0.925	0.843	1.015	0.099
Sedimentation rate	0.969	0.936	1.004	0.086
QRS interval	1.066	1.040	1.093	**<0.001**
fQRS	2.549	1.526	4.259	**<0.001**

CRP: C-reactive protein, fQRS: Fragmented QRS. Statistically significant results (p<0.05) were shown in bold type.

**Table 3:** Baseline characteristics, laboratory and 12-Lead Electrocardiographic parameters of the patients with celiac disease in the fQRS (+) and fQRS (-) subgroups.

	Fragmented QRS (+) (n=68)	Fragmented QRS (-) (n=96)	*P*
Age, years	43 (30.3-50)	39 (25.3-48)	0.227
Sex (Male), n (%)	18 (26.5)	32 (33.3)	0.347
Smoking, n (%)	22 (32.4)	28 (29.2)	0.662
BMI, kg/m^2^	25 (23.4-26)	25.5 (24-27)	0.073
Glucose, mg/dL	91,5 (82-113.8)	93.5 (85-109.3)	0.837
WBC, ×10^3^/ml	7.1 ± 1.7	6.8 ± 1.5	0.409
Hemoglobin, g/dL	13.5 ± 1.7	13.6 ± 1.5	0.496
AST, U/L	18.5 (15-26)	17 (14.3-22)	0.195
ALT, U/L	18 (15.3-23)	21 (16-25.8)	0.065
Creatinine, mg/dL	0.7 (0.6-0.9)	0.7 (0.6-0.8)	0.613
Sodium, mmol/L	140 (138-141.8)	140 (139-141)	0.240
Potassium, mmol/L	4.2 (4-4.5)	4.3 (4.1-4.5)	0.099
Calcium, mg/dL	9.6 (9.1-9.9)	9.5 (9.1-9.7)	0.210
Total cholesterol, mg/dL	172.5 ± 43.8	164.7 ± 35.8	0.211
Triglyceride, mg/dL	124 (80.5-165.3)	119.5 (85-164.5)	0.801
HDL, mg/dL	43 (36-55.8)	44 (36-56.3)	0.405
LDL, mg/dL	97.4 ± 33.7	91.9 ± 30.8	0.288
Sedimentation rate, mm/h	8 (5-13)	8 (5-11)	0.357
C-reactive protein, mg/dL	0.9 (0.5-2.4)	1.1 (0.5-2.9)	0.783
Heart rate, beat/min	78.2 ± 14.3	78.9 ± 11.9	0.712
QRS interval, ms	92 (84.5-96)	92 (86-94)	0.693
QTc interval, ms	423.1 ± 22.2	424.1 ± 24.8	0.796
Disease duration, months	60 (23.5-144)	28.5 (15-71.5)	**0.002**

Results are expressed as: mean ± SD or median (IQR) or frequency (%). SD: Standard deviation, IQR: Interquartile range, BMI: body mass index, WBC: white blood cell, AST: aspartate aminotransferase, ALT: alanine aminotransferase, HDL: high-density lipoprotein, LDL: low-density lipoprotein, QTc: Corrected QT interval. Statistically significant results (p<0.05) were shown in bold type.
